# Self-Management Education for Persons with Parkinson's Disease and Their Care Partners: A Quasi-Experimental Case-Control Study in Clinical Practice

**DOI:** 10.1155/2020/6920943

**Published:** 2020-04-30

**Authors:** Carina Hellqvist, Carina Berterö, Nil Dizdar, Märta Sund-Levander, Peter Hagell

**Affiliations:** ^1^Department of Medical and Health Science, Linköping University, SE-58183 Linköping, Sweden; ^2^Centre for Systems Neurobiology, Department of Biomedical and Clinical Sciences, Linköping University, SE-58183 Linköping, Sweden; ^3^The PRO-CARE Group, Faculty of Health Sciences, Kristianstad University, SE-291 88 Kristianstad, Sweden

## Abstract

**Background:**

Parkinson's disease is a neurodegenerative condition with both physical and mental consequences that affect many aspects of everyday life. Persons with Parkinson's disease and their care partners want guidance from healthcare services in order to develop skills to adjust to life with a long-term condition. The Swedish National Parkinson School is a dyadic self-management programme to support both persons with Parkinson's disease and care partners.

**Objective:**

To assess the outcomes of the Swedish National Parkinson School as reported by participants.

**Design:**

A quasi-experimental case-control study in clinical care using self-reported questionnaires. *Participants.* Swedish National Parkinson School was offered by health care professionals working in clinical care. Participants in the programme were also asked to participate in the study. A matched control group was recruited for a comparison of findings. In total, 92 persons with Parkinson's disease and 55 care partners were included. *Settings*. Five Swedish geriatric and neurologic outpatient clinics.

**Method:**

Data were collected during 2015–2017, before and after participation in the National Parkinson School or before and after seven weeks of standard care. Outcomes were assessed using generic and Parkinson's specific questionnaires. Descriptive statistics were used to describe baseline characteristics. Mann–Whitney *U* and Chi^2^ tests were used to test for between-group differences and within-group differences were tested by the Wilcoxon signed-ranks test.

**Results:**

Improvements regarding health status, constructive attitudes and approaches, and skill and technique acquisition were found after the intervention among persons with Parkinson's disease. No changes were found among care partners.

**Conclusion:**

The findings indicate that the Swedish National Parkinson School may improve health status and self-management among persons with Parkinson's disease, but further studies are needed to better understand the effects of the programme.

## 1. Introduction

Parkinson's disease (PD) is a long-term neurodegenerative condition affecting about 1% of the population over 60 years old. With a longer life expectancy in the general population, the prevalence is expected to double over the coming decades [1–3]. PD is associated with both physical and mental consequences that affect many aspects of daily life [1, 4]. Motor symptoms (i.e., slowing of movements, rigidity, resting tremor, balance, and gait problems) are mainly due to the degeneration of dopaminergic neurons in the brain [1]. However, nonmotor symptoms, such as fatigue, depression, anxiety, cognitive impairment, pain, sleep disturbances, and dysautonomia are also common and add significantly to the burden of living with PD [4, 5]. Symptomatic medical therapy is initially successful but a fluctuating drug response, dyskinesias, and other treatment complications often develop over time [6]. The occurrence and progression of both motor and nonmotor symptoms, often in complex, unpredictable, and fluctuating patterns, have significant consequences in daily life for persons with PD (PwPD) as well as for their family members [7, 8].

Although PwPD need regular healthcare visits, including evaluation of symptoms and adjustment of medical treatment, day-to-day care and management is performed by the persons themselves and their care partners [9, 10]. Accordingly, PwPD and their care partners need to develop emotional, cognitive, and practical skills to adjust to living with the disease. The ability to handle everyday life and deal with uncertainty of the future is vital in order to live well and maintain life satisfaction despite PD [11–14].

Self-care is defined as “the practice of activities that individuals initiate and perform on their own behalf in maintaining life, health and well-being” [15]. For persons affected by long-term conditions, self-care demands change because of disease. The ability to adjust and engage in self-care activities is crucial. Self-care is a wide concept that contains three key components: self-maintenance, self-monitoring, and self-management. Self-maintenance includes activities concerning many aspects of life that a person will have to perform in order to maintain health and well-being [16]. Self-monitoring refers to cognitive processes including observation and assessment of symptoms and activities of daily living leading to self-awareness. Self-management is defined as the ability of a person, in collaboration with family, society, and healthcare services, to handle symptoms, treatments, lifestyle changes, psychosocial strain, and other consequences of disease [17]. All three components are frequently used in the literature concerning people living with long-term disorders, and in nursing practice [18, 19].

A central role of the nursing profession is to provide assistance, support, and advice on health-related issues. Changes in population demographics, with a growing number of persons affected by long-term disorders as well as limited resources and the changing organization of health care have accentuated nurses' need to facilitate and support self-care in the outpatient care setting [20]. In supporting persons with long-term conditions, nurses typically deal with the impact of disease on everyday life as well as medical aspects of the disease [21, 22]. Nurses are therefore considered suitable to guide patients and their families to actively engage in self-care [20, 23–25]. Patient education can be a way to strengthen a person's abilities and boost self-efficacy in becoming actively involved in self-care [26]. Accordingly, nurses themselves acknowledge that their role as an educator is a significant dimension of their profession [24].

Self-management interventions are educational interventions designed to help persons with long-term disorders to deal with the impact of disease in everyday life. To be considered a self-management intervention the education should target cognitive processes and inner motivation and not just provide increased knowledge of the disease and medication. Self-management interventions often include techniques of goalsetting, self-monitoring, problem solving, and action planning [27, 28].

In order to provide self-management education and support to PwPD and their care partners, the European EduPark consortium initiated the development of a standardised educational programme in 2002. This resulted in the self-management programme “Patient Education for Persons with PD and their carers” (PEPP) based on the principles of cognitive behavioural therapy [29]. The purpose is to provide PwPD and their care partners with tools and strategies to increase their ability to manage everyday life with disease and to promote life satisfaction [30]. The programme was tested and evaluated in seven European countries and was found feasible and suitable for most PwPD and care partners [31, 32]. Studies have suggested that PEPP can improve mood and reduce psychosocial strain among both PwPD and care partners [33, 34] and improve self-reported health status among PwPD [35]. These improvements were sustained for three months [36] but returned to baseline after six months [35].

Inspired by PEPP and with self-monitoring as well as self-management as central concepts, the Swedish National Parkinson School (NPS) was developed in 2013 in collaboration between health care professionals, patient representatives, and industry [37, 38] and has since then been implemented in clinical practice across the country. A previous study [39] has indicated that NPS participants experience several benefits, including support from persons in the same situation and improved social connections to family, society, and health care services. Improved knowledge of strategies and cognitive techniques to monitor symptoms and change behaviours, which allowed them to better understand and cope with PD, was also reported. It also increased awareness of the need to adapt a positive mindset and outlook on life and to prioritise activities that promote feelings of well-being and satisfaction with life [39]. However, no formal quantitative outcome assessment of the NPS similar to those of the PEPP has been conducted.

The objective of this study was to assess outcomes of the NPS from the perspective of the participants using self-reported questionnaires regarding, for example, life satisfaction, self-reported health status, emotional well-being, health-directed behaviours, social integration, and support.

## 2. Materials and Methods

### 2.1. Design

This study is a quasi-experimental case-control study of outcomes of the NPS as offered in clinical practice to PwPD and their care partners. In addition, data were also collected from a matched control group.

### 2.2. Participants

All participants were invited to participate in the NPS by health care professionals working at five geriatric and neurologic outpatient clinics across Sweden. The NPS program was already an implemented intervention in the clinical care and it was provided at the participating clinics. Therefore, the researchers had no influence in the invitations or the selection of participants since this was made by the health care professionals. Similarly, the reserachers were not involved in the delivery of the NPS program as it was provided at the participating clinics. Those who agreed to participate in NPS were also informed about the study and contacted by the researchers. The only inclusion criterion applied was a diagnosis of Parkinson's disease for the persons affected. The only exclusion criterion for both persons with Parkinson's disease and care partners was cognitive impairment affecting their ability to understand and respond to the self-reported outcome instruments, as assessed by the health care professionals at the clinic.

**Figure 1:**
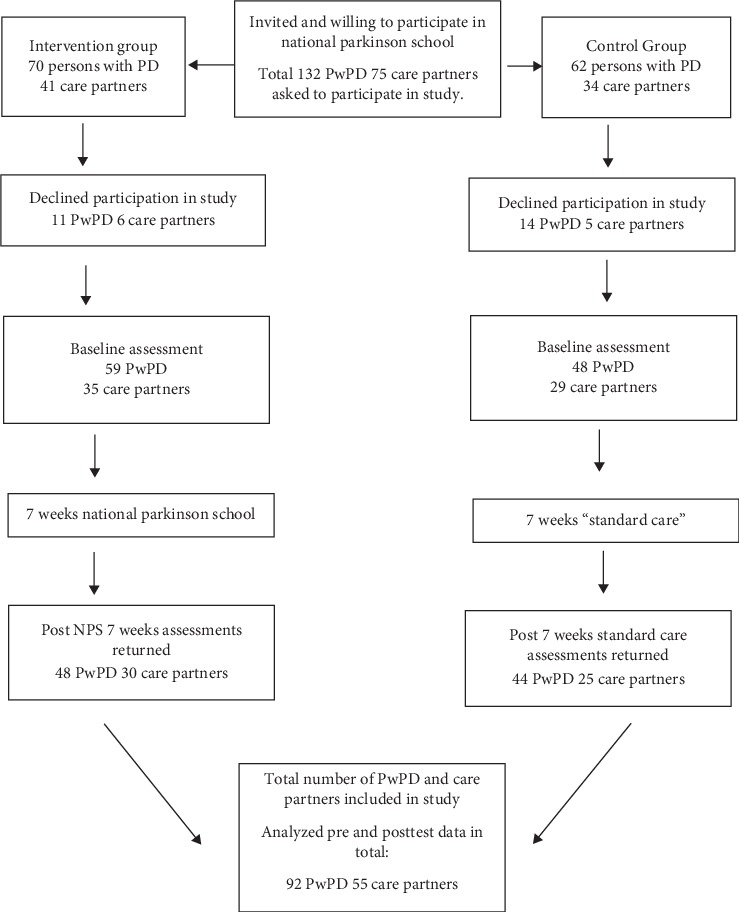
Flow of participants.

An age- and gender matched control group was recruited using patient listings of PwPD cared for at a major university hospital covering a large part of south eastern Sweden according to the same inclusion and exclusion criteria for the intervention group. See Figure 1 for the flow of participants.

### 2.3. Data Collection

Data were collected during 2015–2017. NPS participants were first contacted over telephone by the first author one week before the start of the NPS with information about the study and its purpose. Written information about the study and the questionnaires (see below) were then mailed to the participants with instructions to complete them and bring them to the first NPS session. The questionnaires were collected on-site by the first author immediately before the first NPS session. At that time, the stage of PD according to Hoehn and Yahr was also documented for PwPD [40]. Collecting the questionnaires in person gave the participants an opportunity to ask further questions about the study and clarify any uncertainties they might have had about answering the questionnaires. After the last session (seven weeks later), the same questionnaires were mailed to the participants with instructions to complete and return them within a week. All participants were asked to fill in a short form if there had been any changes in their medication or if there had been any outstanding events making them feel unusually happy or sad recently. The intervention group included 59 PwPD and 35 care partners; 11 PwPD and five care partners dropped out of the NPS before the end of the intervention or did not return follow-up data.

The control group did not participate in the NPS but received standard care. They received written information about the study and those who consented were asked to complete questionnaires and return them by mail to the first author within two weeks. Seven weeks later, they were mailed the same questionnaires again and were asked to complete and return them within two weeks. In total 48 PwPD and 29 care partners agreed to participate in the control group; four in each group did not return follow-up data and were excluded from the study.

Only participants with complete baseline and follow-up assessments were included in the analysis; 48 PwPD and 30 care partners in the intervention group and 44 PwPD and 25 care partners in the control group. See Figure 1 for the flow of participants.

### 2.4. Intervention

**Figure 2:**
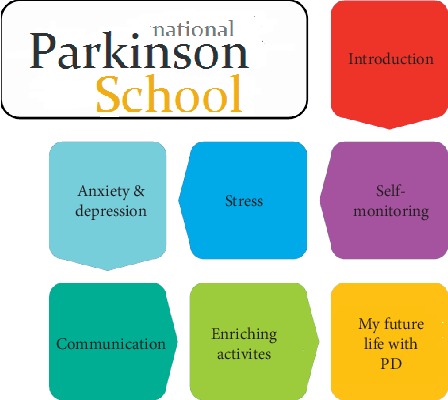
Overview of the themes and topics included in Swedish National Parkinson School.

The NPS is a self-management programme for PwPD and their care partners that aims to provide knowledge and tools to enhance their ability to live and handle life with PD. The programme builds on principles of cognitive behavioural therapy and was developed based on the PEPP [29, 37–39]. The NPS promotes awareness of thoughts, feelings, and actions in relation to the impact of disease on daily life and introduces techniques of self-monitoring in order to provide the knowledge and tools needed to enhance the ability to live and handle life with the disease. It does not primarily focus on the disease itself, but on how to live a good life in the presence of disease. The programme is based on the idea that the participants need both knowledge about the disease itself and an understanding of how it can affect their lives. Having a positive mindset in life and engaging in meaningful and social activities are promoted as ways to enhance satisfaction with life. The NPS consists of seven, two-hour sessions, where PwPD and care partners gather in a small group with a qualified instructor. The instructor is a health care professional experienced with PD and trained to deliver the programme. Each session focuses on a specific topic that is first introduced by the instructor and then followed by group discussions. Each session ends with a 15-minute relaxation exercise. The topic is then applied to the participants' own life situation through practical exercises and homework assignments. The seven themes of the NPS are presented in Figure 2. For more information of the NPS programme, see supporting information file (available here).

### 2.5. Assessments

Data were collected using seven generic and PD specific questionnaires administered before and after participation in the NPS (and at corresponding time points for the control group). The eight-item PD Questionnaire (PDQ-8) was only used by PwPD, and the Zarit Burden Interview (ZBI) only by care partners. The protocol was partly inspired by previous studies investigating outcomes of the PEPP [31, 33–35].

The PDQ-8 is a specific questionnaire regarding perceived PD related health status that was developed as a short form of the PDQ-39 [41]. Each item has five response categories (“never” through “always,” scored 0–4) that are summed and transformed to a 0–100 range where higher scores indicate worse health status.

The EQ-5D is a five-item generic instrument for describing and valuing health states [42]. Each item has three response categories that describe varying levels of problems experienced for each item. It yields a utility value that represents how each combination of responses has been valued by representatives of the general population, from 1 (perfect health) to 0 (dead), as well as negative values representing health states considered worse than death [43]. In this study, we used the experience-based scoring algorithm proposed by Burström et al. [44].

The ZBI is a generic questionnaire designed to evaluate the burden that family caregivers may experience [45]. In this study, we used the 12-item short form of the ZBI [46], which has been found to exhibit psychometric properties that are very similar to those of the original 22-item version when used with care partners of PwPD [47]. Each item has five response categories (“never” through “nearly always,” scored 0–4) that are summed into a total score that may range between 0 and 48, where higher scores indicate higher burden.

The Health Education Impact Questionnaire (heiQ) is a generic instrument designed to evaluate the efficacy of self-management education for persons with long-term disorders [48]. The heiQ consists of 40 items representing eight domains (Positive and active engagement in life, Health-directed activities, skill and technique acquisition, Constructive attitudes and approaches, Self-monitoring and insight, Health service navigation, Social integration and support, emotional distress). Items are scored from 1 (“strongly disagree”) to 4 (“strongly agree”), and total scores within each domain are also between 1 and 4. Higher scores indicate greater self-management, except for the emotional distress domain, where scoring is reversed. A Swedish version of the heiQ has been evaluated among people with various long-term disorders, including PwPD [49]. In addition, the heiQ-Programme evaluation questionnaire was used at follow-up. This questionnaire was designed to evaluate the quality of programme delivery as perceived by participants [48]. In this study, we extended the heiQ-Programme with two questions specific to the NPS, asking the participants whether their expectations of the program had been met and whether it had improved their understanding of PD.

The 11-item Life Satisfaction Checklist (LiSat-11) is a generic questionnaire concerning the respondents' satisfaction with their current life situation as a whole as well as within 10 specific areas [50]. Each area is scored from 1 (“very dissatisfied”) to 6 (“very satisfied”). The questionnaire has previously been used to assess satisfaction with life among PwPD [51, 52].

The 16-item Parkinson Fatigue Scale (PFS-16) was developed to assess fatigue in PwPD but is also considered useful for people who do not have PD [53–55]. Items were scored from 0 (“strongly disagree”) to 4 (“strongly agree”), yielding a possible total score between 0 and 64 (higher scores = more fatigue).

In addition, participants completed a questionnaire on demographic information (age, gender, education, living conditions, etc.), comorbidities, and perceived general health according to item 1 of the RAND-36 questionnaire (“poor” through “excellent,” scored 0–4) [56, 57]. PwPD also rated their difficulties in daily life using the PD Activities of Daily Living Scale (PADLS; “no difficulties” through to “extreme difficulties,” scored 1–5) [58, 59], perceived burden due to PD (“none at all” through to “extreme,” scored 0–4), frequency of memory problems according to item 32 of the PDQ-39 (“never” through to “always,” scored 0–4) [60, 61], and provided information on the presence or absence of motor fluctuations and dyskinesias, time since diagnosis, number of daily medication intakes, and current medical PD therapy.

### 2.6. Sample Size

Sample size was estimated based on the PDQ-8 using G^∗^Power version 3.1.9.4 [62]. Previous studies have estimated the minimal important difference in PDQ-8 scores based on perceived improvement in health status and disease severity to be about 7-8, corresponding to an effect size of around 0.4 [63]. To detect such an effect at an alpha level of 0.05 with 80% power requires a total sample size of at least 35. To compensate for nonconsent, attrition, and uncertainties in estimates we aimed at inviting twice that number of PwPD to participate.

### 2.7. Statistical Analysis

Analyses were performed using IBM SPSS version 23 (IBM Corp., Armonk, NY, USA). Due to the ordinal nature of data and generally nonnormal distributional properties, nonparametric statistics were used. Mann–Whitney *U* and Chi^2^ tests were used to test for between-group differences, while within-group differences (pre-vs. postintervention) were tested by the Wilcoxon signed-ranks test.

### 2.8. Ethical Considerations

This study was approved by the regional ethical review board and conducted in accordance with the guidelines in the Declaration of Helsinki [64]. Participants received written and oral information about the study and written consent were collected from all participants.

## 3. Results

### 3.1. Attrition

In the intervention group, there was a total of 11 PwPD and five care partners without posttest data. Reasons included nonreturn of follow-up questionnaires (PwPD, *n* = 2; care partners, *n* = 1) and dropout from the programme due to dissatisfaction with the programme's group format (PwPD, *n* = 1; care partners, *n* = 1), health issues (PwPD, *n* = 3; care partners, *n* = 2), and unknown reasons (PwPD, *n* = 5; care partners, *n* = 1). In the control group, four PwPD and four care partners did not return their follow-up questionnaires.

### 3.2. Background Characteristics

The background characteristics of all participants are reported in Table 1 and did not differ between intervention and control groups except for a larger proportion of male participants in the intervention group. Age varied between 32 and 85 years in the whole sample with a median age between 68 and 72 in the various groups. PwPD reported between 1 and 11 daily medicine intakes (median, four times/day) and having had their PD diagnosis since 0–30 years with a median of five and seven years in the intervention and control groups, respectively. Hoehn and Yahr stages of PD varied between I and IV (median, III) in the intervention group.

### 3.3. Outcomes among Persons with Parkinson's Disease

At baseline (Table 2), PwPD in the control group scored higher on the LiSat-11 domain Life as a whole (*P*=0.031) and the heiQ domain skill and technique acquisition (*P*=0.002) than those in the intervention group. These differences were no longer present at follow-up (Table 2).

Following participation in the NPS (Table 2), PwPD reported improved health as indicated by both PDQ-8 (*P*=0.028) and EQ5D (*P*=0.023) scores. Additionally, there were improvements in constructive attitudes and approaches (*P*=0.003) and skill and technique acquisition (*P* < 0.001) of the heiQ. The pattern of changes in other outcomes also suggested improvements following participation in the NPS, but these failed to reach statistical significance (Table 2).

In contrast, with the exception of emotional distress (heiQ), outcomes in the control group exhibited a pattern of deterioration over seven weeks of standard care (Table 2). Among these, satisfaction with life as a whole (*P*=0.011), leisure (*P*=0.028), and contacts (*P*=0.013) (LiSat-11) reached significance.

### 3.4. Outcomes among Care Partners

There were no statistically significant differences between the intervention and control groups of care partners (Table 3). Scores in the care partners intervention group tended to show slight improvements or remained stable following participation in the NPS but did not reach levels of statistical significance. In the control group, most scores stable or slightly worse following seven weeks of standard care; of these, only satisfaction with life as a whole (LiSat-11) was significantly lower (*P*=0.035) after seven weeks of standard care (Table 3).

### 3.5. Programme Evaluation

The heiQ-Programme evaluation questionnaire showed median values representing high levels of satisfaction with the NPS among the PwPD as well as care partners (Table 4). For example, both PwPD and care partners agreed that participating in the NPS was worthwhile, that its content was relevant, and that their understanding of PD had improved. There was a nonsignificant trend (*P*=0.073) for care partners to find the NPS more helpful than PwPD in terms of goal setting.

### 3.6. Self-Reported Confounding Factors

PwPD and care partners reported some factors that occurred between baseline and follow-up that might have had an impact on their mood and health. These included health problems or deaths in the family (*n* = 3), own health issues (*n* = 3), improved health (*n* = 3), birth of grandchildren (*n* = 4), and changes in medication (*n* = 10). These factors were reported and distributed in both the intervention and control groups. Individual comparison of the assessments of persons reporting possible confounders did not imply that their answers had been influenced and were not extreme in any way.

## 4. Discussion

This study aimed to evaluate the effects of the self-management programme NPS for PwPD and their care partners from the perspective of the participants. The results suggest that participation in the programme is associated with improvements in self-assessed health status and in self-management abilities among PwPD. Although most outcomes did not reach statistical significance, the general pattern reflected improvements in the intervention group, whereas the control group tended to worsen or remain stable. A similar but less obvious pattern was found for care partners.

Our findings revealed improved perceived PD related as well as general health status among PwPD following NPS participation. This is in line with previous results reported for the PEPP [34, 35], suggesting that benefits associated with the PEPP were retained despite the contextual adaptations that were made when developing the NPS. In parallel with unaltered health status in the control group of PwPD there was a deterioration in life satisfaction, which in turn tended to improve in the intervention group. While the reasons for this remain hypothetical, one possibility is that the lack of health benefits and unaltered outlook and coping skills may have played a role in decreasing satisfaction with life. However, this cannot be determined based on the data from the present study.

We found improvements in two domains of the heiQ: constructive attitudes and approaches and skill and technique acquisition. Improvements in the former domain reflect a shift in how persons view the impact of disease in their everyday lives and are connected to a mindset of not allowing disease to control their lives. Improvements in skill and technique acquisition reflect better knowledge of skills and techniques to manage and cope with the impact of PD, including symptoms and problems in everyday life [48]. This is in line with the intentions of the NPS. Previous studies have found that PwPD and care partners wish to acquire the knowledge and skills needed to improve their self-care abilities and their interaction with health care services through shared decision-making [65, 66]. The improvements according to the heiQ found here are therefore encouraging and suggest that the NPS can help PwPD acquire the necessary knowledge and skills to facilitate active self-care behaviours and participation in decisions regarding their care.

This study did not find any significant changes associated with participation in the NPS among care partners. This might be due to the selection of instruments. For example, we used the generic ZBI to assess burden, which may have been too nonspecific to capture changes among care partners of PwPD. In contrast to our findings, previous evaluations of the PEPP reported improvements in care partners' psychosocial strain and need for help, as assessed using the BELA-A-k questionnaire [33, 34]. Since the BELA-A-k was developed specifically for care partners of care partners [67], it might be more suitable for capturing changes related to PD related strain. However, the BELA-A-k is not available for use in Sweden. Nevertheless, similar to the results from the PEPP [33], care partners in this study expressed positive experiences from the NPS, as indicated by generally high scores on the heiQ-Programme evaluation questionnaire. This supports the value of the intervention, although the outcome assessment tools were unable to reflect any improvements. It should also be noted that previous studies have highlighted the importance of care partners being involved and having knowledge of PD to be able to plan for the future, maintain mutuality in relationships, and have a good quality of life [68]. The support of a care partner is connected to higher levels of self-efficacy to engage in self-care activities and a stronger sense of coherence among PwPD [22, 69].

The selection of outcome measures in this study was partly influenced by previous studies of the PEPP [31, 33–35]. The reason for doing so was to investigate if the NPS, which is influenced by the PEPP, showed similar results to those described by A'Campo and colleagues. However, it may be questioned whether these outcomes are the most appropriate ones for evaluating the effects of this type of intervention. Therefore, we also added the heiQ, which targets specific outcomes of self-management interventions rather than aspects that might also be influenced by other circumstances in life [48]. In this perspective, our observations suggest that the heiQ is suitable and sensitive enough to detect effects of self-management interventions among PwPD and should also be considered in future studies. However, there may also be other outcomes that should be considered. For example, studies investigating outcomes of self-management interventions among persons with other long-term conditions have assessed aspects such as self-efficacy, coping, sense of coherence, and self-management of daily activities [70], that may be appropriate to include in future studies of the NPS to further improve understanding of programme effects. Indeed, one study of the PEPP included several such outcomes and found beneficial effects regarding coping [36]. Furthermore, depressive symptoms are common in PD and can affect function and thereby the effect of self-management interventions as well as influencing responses to questionnaires, adding a tool to assess depression should be considered [71, 72]. Finally, the NPS is designed as a dyadic intervention intended to promote self-management and coping in the context of shared everyday life. Therefore, assessing possible effects on mutuality could also be of interest to understand the impact of the programme [68].

The current study design prevents any conclusions regarding the long-term effects of the NPS. However, previous studies have shown that effects of this type of intervention can last up to three months among PwPD [35, 36]. Assessment of long-term effects that can be influenced by other factors than educational interventions is difficult and the risk of drawing false conclusions in either direction is apparent [48]. In addition, with a progressive disease such as PD it is known that as symptom burden increases, satisfaction with life will decline for both persons affected and their care partners [52, 73, 74]. This impact due to progression of the disease itself should be considered when evaluating long-term outcomes. If the long-term effects of self-management interventions for PwPD are investigated, a reasonable timeframe and alternative outcome assessment tools will be needed.

As this study was designed to reflect the outcomes of the NPS in clinical care, the selection of participants was not restricted by narrow selection criteria. Instead, health care professionals invited persons to the programme based upon their clinical judgement, for example, persons in a certain stage of disease or with an expressed interest in these types of interventions. Accordingly, participants showed a relatively large degree of heterogeneity in background characteristics. While this may have introduced a selection bias and limited the possibilities to detect changes in outcomes, it may also better reflect clinical reality.

Finally, it may be argued that the sample size was insufficient to allow fair detection of programme effects, particularly among care partners. While a more even distribution of PwPD and care partners would have been desirable, the current ratio appears reflective of clinical reality. Furthermore, our sample size estimation was based on an outcome measure previously used as a primary end-point among PwPD [34], not care partners, since the primary care partner outcome measure in previous studies was not available for use in this study. The results of this study for care partners should therefore be interpreted with some caution. A larger sample of PwPD in the intervention group would also have allowed for subgroup analyses, for example, regarding disease severity. This could have added valuable information regarding when the NPS is most useful to participants and may therefore be of value to consider in future research. For example, a previous qualitative study on the NPS indicated that PwPD value different programme components differently depending onstage and duration of disease [39]. In addition, future studies should also consider health economic aspects to better understand the full value and implications of the NPS.

## 5. Conclusions

The NPS is the first structured attempt in the Swedish healthcare to provide a uniform patient education and self-management model for PwPD and their care partners. This study shows that the programme can improve health and the skills required to handle disease and manage symptoms in everyday life and can strengthen the mindset of persons affected by PD of being in charge and not allowing disease to control life. Although we failed to detect any effects among care partners, the NPS was considered at least as valuable by care partners as by PwPD. These results are encouraging and illustrate that the programme is beneficial and valuable for participants. The NPS should therefore be offered as an integrated part of a holistic person-centred standard care, and resources should be allocated for the provision of the programme. Further studies are needed to better understand the effects of the NPS on participants as well as the impact on health care utilisation and organization.

## Figures and Tables

**Table 1:** Background characteristics^*a*^.

	Persons with Parkinson's disease			Care partners		
	Intervention group (*n* = 59)	Control group (*n* = 48)	*P*-value	Intervention group (*n* = 35)	Control group (*n* = 29)	*P*-value
Age	71 (65–75)	68 (64–75)	0.506^*b*^	72 (68–77)	69 (67–74)	0.112^*b*^
Male gender, *n* (%)	24 (41)	32 (67)	0.007^*c*^	22 (63)	7 (24)	0.002^*c*^

Education, *n* (%)						
Primary school (9 years)	15 (25)	8 (17)	0.540^*c*^	12 (34)	5 (17)	0.328^*c*^
High school (11–13 years)	19 (32)	18 (38)		8 (23)	7 (24)	
University	25 (42)	22 (46)		15 (43)	16 (55)	

Living with someone	52 (88)	41 (85)	0.678^*c*^	34 (97)	28 (97)	0.892^*c*^
Relation to person with PD, *n* (%)						
Spouse	—	—	—	34 (97)	28 (97)	1.000^*c*^
Son/daughter	—	—		1 (3)	1 (3)	

Housing, *n* (%)						
Own house	33 (56)	32 (67)	0.162^*c*^	—	—	—
Own apartment	15 (25)	13 (27)		—	—	
Rental apartment	11 (19)	3 (6)		—	—	

Comorbidity, *n* (%)	31 (53)	27 (60)	0.506^*c*^	19 (54)	17 (59)	0.728^*c*^
Perceived general health^*d*^	1 (1–2)	2 (1–2)	0.242^*b*^	2 (2–3)	2 (2–3)	0.915^*b*^
PADLS^*e*^	2 (1–2)	2 (2–2)	0.918^*b*^	—	—	—
Hoehn and Yahr stage of PD^*f*^	3 (2–3)	—	—	—	—	—
Time since diagnosis (years)	5 (2–7)	7 (3–8)	0.147^*b*^	—	—	—
Perceived PD burden^*g*^	2 (2–3)	2 (2–3)	0.829^*b*^	—	—	—
Memory problems in last 30 days^*h*^	2 (1–2)	2 (1–2)	0.450^*b*^	—	—	—
Number of medication times/day	4 (3–5)	4 (3–6)	0.984^*b*^	—	—	—
Motor fluctuations, *n* (%)	30 (51)	33 (69)	0.061^*c*^	—	—	—
Dyskinesias, *n* (%)	17 (29)	21 (45)	0.091^*c*^	—	—	—

PD medications, *n* (%)						
Levodopa	54 (95)	45 (94)	1.000^*c*^	—	—	—
Dopamine agonists	31 (54)	34 (71)	0.084^*c*^	—	—	—
COMT inhibitors	11 (19)	8 (17)	0.727^*c*^	—	—	—
MAO-B inhibitors	23 (39)	22 (46)	0.572^*c*^	—	—	—
Other^*i*^	2 (4)	2 (4)	1.000^*c*^	—	—	—

Advanced treatment, *n* (%)						
Deep brain stimulation	1 (2)	2 (4)	0.586^*c*^	—	—	—
S.c. apomorphine infusion	1 (2)	1 (2)	1.000^*c*^	—	—	—
Levodopa/carbidopa intestinal gel	4 (7)	1 (2)	0.372^*c*^	—	—	—

^*a*^Data are median (q1-q3) unless otherwise noted. ^*b*^Mann–Whitney *U* test. ^*c*^Chi-Square test or Fisher's exact test, as appropriate. ^*d*^Possible scores, 0–4 (0 = Poor, 4 = Excellent). ^*e*^Possible scores, 1–5 (1 = No difficulties, 5 = Extreme difficulties). ^*f*^Possible stages, I–V (I = Mild unilateral disease, V = Confined to bed or wheelchair unless aided). ^*g*^Possible scores, 0–4 (0 = None at all, 4 = Extreme). ^*h*^Possible scores, 0–4 (0 = Never, 4 = Always). ^*i*^Other medications included amantadine (*n* = 2), anticholinergics (*n* = 1), clozapine (*n* = 1). PADLS: Parkinson's disease Activities of Daily Living Scale; PD: Parkinson's disease; COMT: Cathechol-O-Methyl Transferase; MAO-B: Mono Amine Oxidase type B; s.c.: subcutaneous.

**Table 2:** Differences between and in persons with Parkinson's disease before and after seven weeks of participation in the National Parkinson School (intervention group) and after seven weeks of ordinal care (control group)^*a*^.

		Intervention group (*n* = 48)	Control group (*n* = 44)	*P*-value^*b*^
Health status (PDQ-8)^*d*^	Baseline	28.1 (17.2–39.1)	25 (12.5–37.5)	0.301
	Follow-up	23.4 (14.8–37.5)	23.4 (13.3–37.5)	0.713
	*P*-value^*c*^	**0.028**	0.644	

Health valuation (EQ5D)^*e*^	Baseline	0.87 (0.71–0.93)	0.86 (0.79–0.93)	0.473
	Follow-up	0.88 (0.78–0.93)	0.86 (0.79–0.91)	0.279
	*P*-value^*c*^	**0.023**	0.866	

Health education impact (heiQ)^*f*^				
Health-directed activities	Baseline	3.25 (2.75–3.75)	3.25 (2.75–3.75)	0.865
	Follow-up	3.25 (2.75–3.75	3 (2.75–3.50)	0.344
	*P*-value^*c*^	0.323	0.437	

Positive and active engagement in life	Baseline	2.8 (2.6–3.2)	3 (2.6–3.4)	0.088
	Follow-up	3 (2.8–3.2)	3 (2.6–3.4)	0.945
	*P*-value^*c*^	0.058	0.150	

Emotional distress	Baseline	2 (1.5–2.67)	2.17 (1.83–2.67)	0.529
	Follow-up	2 (1.5–2.46)	1.83 (1.33–2.5)	0.536
	*P*-value^*c*^	0.436	**0.020**	

Self-monitoring and insight	Baseline	2.83 (2.58–3.17)	3 (2.5–3.17)	0.445
	Follow-up	3 (2.71–3.17)	3 (2.67–3.17)	0.652
	*P*-value^*c*^	0.572	0.285	

Constructive attitudes and approaches	Baseline	3 (2.6–3.2)	3 (2.8–3.6)	0.075
	Follow-up	3 (2.8–3.4)	3.2 (2.8–3.6)	0.971
	*P*-value^*c*^	**0.003**	0.845	

Skills and techniques acquisition	Baseline	2.5 (2–2.75)	2.88 (2.5–3.19)	**0.002**
	Follow-up	3 (2.5–3)	2.75 (2.44–3)	0.463
	*P*-value^*c*^	**<0.001**	0.464	

Social integration and support	Baseline	3 (2.4–3.6)	3 (2.8–3.4)	0.561
	Follow-up	3 (2.8–3.6)	3 (2.6–3.4)	0.389
	*P*-value^*c*^	0.110	0.324	

Health service navigation	Baseline	2.9 (2.4–3.2)	3 (2.8–3.2)	0.112
	Follow-up	3 (2.6–3.6)	3 (2.8–3.4)	0.903
	*P*-value^*c*^	0.286	0.368	

Life satisfaction (LiSat-11)^*g*^				
Life as a whole	Baseline	4 (4–5)	5 (4–5)	**0.031**
	Follow-up	4.5 (4–5)	4.5 (4–5)	0.868
	*P*-value^*c*^	0.117	**0.011**	

Vocation	Baseline	4 (3–5)	4 (4–5)	0.078
	Follow-up	4 (4–5)	4.5 (4–5)	0.170
	*P*-value^*c*^	0.159	0.157	

Economy	Baseline	5 (4–5)	5 (5–5)	0.077
	Follow-up	4 (4–5)	5 (4–5)	**0.042**
	*P*-value^*c*^	0.221	0.489	

Leisure	Baseline	4 (4–5)	5 (4–5)	0.105
	Follow-up	4 (4–5)	5 (4–5)	0.889
	*P*-value^*c*^	1.000	**0.028**	

Contacts	Baseline	5 (4–5)	5 (4–5)	0.472
	Follow-up	5 (4–5)	4 (4–5)	0.481
	*P*-value^*c*^	0.815	**0.013**	

Sexual life	Baseline	3 (2–4)	3 (2–4)	0.314
	Follow-up	3 (2–4)	3 (2–5)	0.364
	*P*-value^*c*^	1.000	0.414	

ADL	Baseline	5 (4–5)	5 (4–6)	0.289
	Follow-up	5 (4–5)	5 (4–6)	0.401
	*P*-value^*c*^	0.168	0.186	

Family life	Baseline	5 (4–6)	5 (4–6)	0.203
	Follow-up	5 (4–6)	5 (5–6)	0.385
	*P*-value^*c*^	0.729	0.712	
Partner relationship	Baseline	5 (4–6)	6 (4.5–6)	0.266
	Follow-up	5 (4.5–6)	5 (5–6)	0.841
	*P*-value^*c*^	0.696	0.244	

Somatic health	Baseline	4 (3–4)	4 (4–5)	0.068
	Follow-up	4 (3–4)	4 (3–5)	0.930
	*P*-value^*c*^	0.263	0.054	

Psychological health	Baseline	4 (4–5)	5 (4–5)	0.367
	Follow-up	4 (4–5)	5 (4–5)	0.088
	*P*-value^*c*^	0.442	0.536	

Fatigue (PFS-16)^*h*^	Baseline	28 (19–38.8)	32 (22–38)	0.814
	Follow-up	32 (20–38)	27 (16–40)	0.946
	*P*-value^*c*^	0.164	0.832	

^*a*^Data are median (q1-q3). ^*b*^Mann–Whitney *U* test for comparisons between intervention and control groups. ^*c*^Wilcoxon's signed rank test for comparisons between baseline and follow-up within intervention and control groups. ^*d*^Possible scores, 0–100 (100 = worse health status). ^*e*^Possible scores, 0–1 (1 = better valued health state). ^*f*^Possible stages, 1–4 (4 = better; except for emotional well-being, where 1 = better). ^*g*^Possible scores, 1–6 (6 = higher life satisfaction). ^*h*^Possible scores, 0–64 (64 = more fatigue). PDQ-8: the eight-item Parkinson's Disease Questionnaire; EQ5D: the five-dimensional EuroQol Questionnaire; heiQ: Health Education Impact Questionnaire; LiSat-11: the 11-item Life Satisfaction Checklist; PFS-16: the 16-item Parkinson Fatigue Scale.

**Table 3:** Differences between care partners and persons with Parkinson's disease before and after seven weeks of participation in the National Parkinson School (intervention group) and after seven weeks of ordinal care (control group)^*a*^.

		Intervention group (*n* = 30)	Control group (*n* = 25)	*P*-value^*b*^
Health valuation (EQ5D)^*d*^	Baseline	0.93 (0.88–0.97)	0.93 (0.88–0.97)	0.508
	Follow-up	0.97 (0.88–0.97)	0.93 (0.88–0.97)	0.251
	*P*-value^*c*^	0.837	0.944	

Life satisfaction (LiSat-11)^*e*^				
Life as a whole	Baseline	5 (4–5)	5 (4–6)	0.302
	Follow-up	5 (4–5)	5 (4–6)	0.802
	*P*-value^*c*^	0.414	**0.035**	

Vocation	Baseline	5 (4–5)	5 (4–5.75)	0.388
	Follow-up	5 (4.25–5)	5 (4–5)	0.261
	*P*-value^*c*^	0.655	0.109	

Economy	Baseline	5 (5–5)	5 (5–5.25)	0.515
	Follow-up	5 (4–5.25)	5 (4–5)	0.945
	*P*-value^*c*^	0.317	0.132	

Leisure	Baseline	5 (4–5)	5 (4.75–5)	0.072
	Follow-up	5 (4–5)	5 (4–5)	0.964
	*P*-value^*c*^	0.248	0.206	

Contacts	Baseline	5 (5–5)	5 (4–5.25)	0.666
	Follow-up	5 (4–5)	5 (4–6)	0.671
	*P*-value^*c*^	0.366	0.564	

Sexual life	Baseline	4 (2–5)	4 (3.5–5)	0.509
	Follow-up	4 (3–5)	4 (2.25–5)	0.776
	*P*-value^*c*^	0.491	0.417	

ADL	Baseline	6 (5–6)	6 (5–6)	0.586
	Follow-up	6 (5–6)	6 (5.25–6)	0.486
	*P*-value^*c*^	0.891	0.317	

Family life	Baseline	5 (4–6)	5.5 (5–6)	0.227
	Follow-up	6 (5–6)	6 (5–6)	0.801
	*P*-value^*c*^	0.290	0.655	

Partner relationship	Baseline	6 (5–6)	6 (5–6)	0.773
	Follow-up	6 (5–6)	6 (5–6)	0.792
	*P*-value^*c*^	0.763	0.705	

Somatic health	Baseline	5 (4–5)	5 (4–5)	0.805
	Follow-up	5 (5–5)	5 (4–5)	0.659
	*P*-value^*c*^	0.739	0.655	

Psychological health	Baseline	5 (5–6)	5 (4.75–6)	0.371
	Follow-up	5 (5–6)	5 (4.5–6)	0.851
	*P*-value^*c*^	0.279	0.180	

Fatigue (PFS-16)^*f*^	Baseline	9 (0–17.75)	5 (1–19)	0.661
	Follow-up	6 (0–20)	11.5 (1.25–25)	0.296
	*P*-value^*c*^	0.678	0.138	

Caregiver burden (ZBI-22)^*g*^	Baseline	7 (3–13)	6 (0.75–12.5)	0.495
	Follow-up	8 (3.25–12.75)	5 (2–13.25)	0.659
	*P*-value^*c*^	0.090	0.548	

^*a*^Data are median (q1-q3). ^*b*^Mann–Whitney *U* test for comparisons between intervention and control groups. ^*c*^Wilcoxon's signed rank test for comparisons between baseline and follow-up within intervention and control groups. ^*d*^Possible scores, 0-1 (1 = better valued health state). ^*e*^Possible scores, 1–6 (6 = higher life satisfaction). ^*f*^Possible scores, 0–64 (64 = more fatigue). ^*g*^Possible scores, 0–48 (48 = more burden). EQ5D: the 5-dimensional EuroQol questionnaire; LiSat-11: the 11-item Life Satisfaction Checklist; PFS-16: the 16-item Parkinson Fatigue Scale; zbi-22: the 22-item Zarit Burden Interview.

**Table 4:** Results of the heiQ-Program evaluation among persons with Parkinson's disease and care partners after participation in the National Parkinson School^*a*,*b*^.

Items (abridged)	Persons with Parkinson's disease (*n* = 48)	Care partners (*n* = 26)	*P*-value^*e*^
I will tell people that the NPS is very worthwhile^*b*^	5 (4–5)	5 (4.75–5)	0.349
The NPS has helped me set reasonable and achievable goals^*b*^	4 (4–5)	5 (4–5)	0.073
I trust the information and advice given in the NPS^*b*^	5 (4–6)	5 (5–5)	0.649
NPS leaders were very well-organized^*b*^	5 (4–5)	5 (4–6)	0.091
It was worth my time and effort to take part in the NPS^*b*^	5 (4–6)	5 (5–6)	0.405
Difficult topics and discussions were handled well^*b*^	5 (4–5)	5 (5–6)	0.136
NPS content was very relevant to my situation^*b*^	5 (4–5)	5 (4–5)	0.309
Everyone had the chance to speak if they wanted to^*b*^	5 (5–6)	5.5 (5–6)	0.425
The group worked very well together^*b*^	5 (4–5)	5 (4.5–5.5)	0.158
My understanding of PD has improved^*c*,*d*^	3 (2–3)	3 (2–3)	0.283
My expectations of the NPS were met^*c*,*d*^	3 (2–3)	3 (2–3)	0.177

^*a*^Data are median (q1-q3). ^*b*^Possible scores, 1–6 (1 = Totally disagree, 6 = Totally agree). ^*c*^Study specific items not included in the original heiQ-Program evaluation questionnaire. ^*d*^Possible scores, 1–3 (1 = Disagree, 3 = Agree). ^*e*^Mann–Whitney *U* test.

## Data Availability

The data used to support the findings of this study are restricted by the Regional Ethical Review Board of Linköping, Sweden, in order to protect patient privacy. Data are available from the corresponding author for researchers who meet the criteria for access to confidential data.

## Supplementary Materials

Supplementary Materials Flow of participants.
